# A practical guide to managing cardiopulmonary toxicities of tyrosine kinase inhibitors in chronic myeloid leukemia

**DOI:** 10.3389/fmed.2023.1163137

**Published:** 2023-05-05

**Authors:** Rasha Kaddoura, Wafer A. Dabdoob, Khalid Ahmed, Mohamed A. Yassin

**Affiliations:** Hamad Medical Corporation, Doha, Qatar

**Keywords:** chronic myeloid leukemia, tyrosine kinase, heart failure, pulmonary arterial hypertension, QT prolongation, dasatinib, ponatinib, pleural effusion

## Abstract

Tyrosine kinase inhibitors (TKIs) have revolutionized the treatment of chronic myeloid leukemia (CML) but their use was associated with a range of serious cardiopulmonary toxicities including vascular adverse events, QT prolongation, heart failure, pleural effusion, and pulmonary arterial hypertension. Dedicated clinical management guidelines for TKI-induced toxicities are not available. This review aims to discuss TKI-associated cardiopulmonary toxicities and proposes a practical guide for their management.

## Introduction

**Figure 1:**
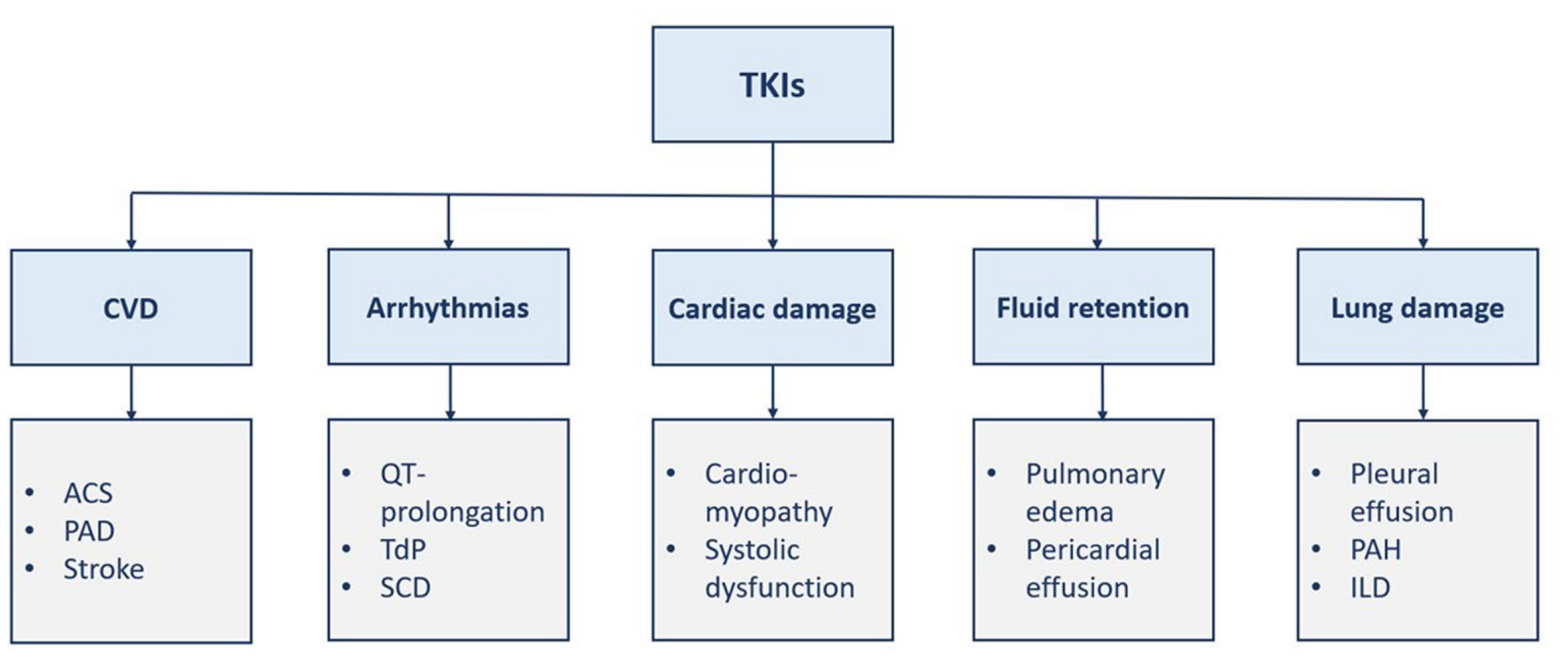
Manifestations of TKI-induced cardiopulmonary toxicities. ACS, acute coronary syndrome; CVD, cardiovascular disease; ILD, interstitial lung disease; PAD, Peripheral arterial disease; PAH, pulmonary arterial hypertension; SCD, sudden cardiac death; TdP, torsade de pointes; TKI, tyrosine kinase inhibitor(s).

Protein kinases are enzymes involved in different signaling pathways and regulate various cellular functions such as proliferation, differentiation, and death (1, 2). Thus, carcinogenesis can be triggered by disturbances in such processes. Kinase inhibitors have brought about a paradigm shift in the treatment of many diverse malignancies (1). Chronic myeloid leukemia (CML), a rare myeloproliferative disease, is associated with chromosomal translocation (i.e., Philadelphia chromosome), which encodes BCR::ABL1 oncoprotein, through the fusion of BCR and ABL1 genes, with active tyrosine kinase activity (3–6). Thus, tyrosine kinase inhibitors (TKIs) are currently the cornerstone treatment of CML (6). TKIs are categorized into small-molecule TKIs and monoclonal antibody drugs. The latter group inhibits proliferation, angiogenesis, and invasion of tumor cells. The former group is less selective than the other one and may cause more adverse events. Different TKIs, namely imatinib, dasatinib, nilotinib, bosutinib, and ponatinib, are approved in Europe and the United States for the treatment of CML. Although the five TKIs inhibit BCR::ABL1, they differ in their targeted site, mechanisms, efficacy, and safety. Cardiovascular toxicities of TKIs have several manifestations such as heart failure, arrhythmias, and fluid retention (1, 2). However, it is hard to distinguish between the cardiovascular events caused by TKIs from the events that occur due to the patient’s risk factors (7). Vascular adverse events and pulmonary toxicities are also associated with TKI therapy (Figure 1) (3, 8). Herein, this review discusses the TKI-associated cardiopulmonary toxicities, including their mechanisms of toxicity, and proposes a practical guide to their recognition, management, and monitoring.

## Tyrosine kinase inhibitors

Tyrosine kinase inhibitors have revolutionized the treatment of CML with the first-generation agent, imatinib (4, 9). The subsequent more effective second-generation agents (bosutinib, dasatinib, and nilotinib) were developed to overcome imatinib resistance (4, 8, 9). However, both generations are highly resistant in the presence of BCR::ABL1T315I mutation, which leads to rapid disease progression and limited mortality improvement. Consequently, the third-generation ponatinib was developed and approved in patients with BCR::ABL1T315I mutation (6, 9). Table 1 summarizes the general characteristics of TKIs (1–3, 8, 10). The three additional third-generation agents have been investigated in TKI-resistant CML and included olverembatinib, asciminib, and vodobatinib. Olverembatinib (HQP1351) was well-tolerated and effective in TKI-resistant chronic and accelerated phase CML, particularly in the presence of BCR::ABL1T315I mutation (9). It has been granted a fast-track designation for CML in the United States (May 2020) and a breakthrough therapy designation for CML in the European Union and China (November 2021) (11). Although the first approval of asciminib was received in October 2021 (United States) for Philadelphia chromosome-positive CML, there are several ongoing trials investigating new indications as monotherapy in chronic phase CML patients with or without T315I mutation previously treated with two or more TKIs or as add-on therapy to other TKIs (12). In a dose-escalating Phase I study, vodobatinib showed encouraging efficacy in both ponatinib-treated and ponatinib-naïve patients with chronic phase CML (13). An innovative fourth-generation agent, PF-114, that structurally resembles ponatinib can inhibit wild-type and mutated BCR::ABL (14, 15). PF-114, unlike Ponatinib, does not inhibit the vascular endothelial growth factor receptor (VEGFR), hence having the benefit of reducing cardiovascular adverse effects (15). Table 2 presents the general characteristics of the novel TKIs (9, 11, 12, 15).

**Table 1:** The general characteristics of tyrosine kinase inhibitors in chronic phase chronic myeloid leukemia.

Generation	First generation	Second generation			Third generation
Agents	Imatinib	Dasatinib	Nilotinib	Bosutinib	Ponatinib
Approval year (EMA/FDA)	2001/2003	2006/2006	2007/2007	2013/2012	2013/2012
Potency versus imatinib	–	300 times	30 times	45 times	500 times
Molecular targets	BCR-ABL, c-KIT, CSF-1R, PDGFR-α, −β, DDR-1, −2	BCR-ABL, c-KIT, SFKS, PDGFR- α, −β	BCR-ABL, CSF-1R, c-kit, DDR, PDGFR- α, −β	BCR-ABL, c-KIT, Hck, PDGF, Src	BCR-ABL, c-KIT, EGFR, PDGFR, VEGFR
First line TKIs	Yes	Yes	Yes	No	No
Second line TKIs					
Intolerance to 1st line	Yes	Yes	Yes	Yes	No
Failure of 1st line imatinib	–	Yes	Yes	Yes	Yes
Failure of 1st line dasatinib or nilotinib	No	Yes	Yes	Yes	Yes
Third line TKIs*	Any not tried TKI				
Daily dose (chronic phase)	400–800 mg	100 mg	300–400 mg twice	400–600 mg	45 mg
Metabolism (CYP enzyme)	CYP3A4 Minor metabolism: CYP1A2, CYP2D6, CYP2C9, CYP2C19	CYP3A4	CYP3A4	CYP3A4	CYP3A4, CYP3A5, CYP2C6, CYP2C8
Cardiopulmonary adverse events	Pleural effusion	VAE, QT prolongation, PH, pleural/ pericardial effusion, HTN, HF	VAE, QT prolongation, AF, HTN, hyperlipidemia, hyperglycemia, IHD, CVA	VAE, pleural/pericardial effusion, HTN	VAE, HTN, IHD, CVA, AF, HF, venous thrombosis, PH
Other non-hematological adverse events	Diarrhea, Hepatobiliary and skin disorders	Hepatobiliary disorders, hemorrhage	Hepatobiliary, skin, metabolism, and nutrition disorders	Diarrhea, Hepatobiliary and skin disorders	Diarrhea, hepatobiliary, and skin disorders
Preferred in	CVD, DM, PAD, PAH	DM, (PAD?)	PAH	CVD, DM, PAD, PAH	–
Less preferred in	–	CVD, PAH	CVD, DM, PAD	–	PAH

*Intolerance and/or failure 2nd line TKI.

CML, chronic myeloid leukemia; CSF1R, colony stimulating factor 1 receptor; CSK, C-terminal src kinase; CVA, cerebrovascular accident (ischaemic); CYP, cytochrome P450; DDR1, discoidin domain receptor 1; EGFR, epidermal growth factor receptor; EMA, European Marketing Authorization; FDA, Food and Drug Administration; IHD, ischaemic heart disease; PAH, pulmonary arterial hypertension; HCK, haemopoeitic cell kinase; PDGFR, platelet-derived growth factor receptor; TKI(s), tyrosine kinase inhibitor(s); VAE, vascular adverse events; VEGFR, vascular endothelial growth factor receptor.

**Table 2:** The general characteristics of novel tyrosine kinase inhibitors.

Generation	Third generation			Fourth generation
Agents	Olverembatinib	Asciminib	Vodobatinib	PF-114
Alternative names	APG 1351; D 824; GZD 824; HQP 1351	ABL 001; ABL-001; STAMP inhibitor	K0706	–
Approval	US: fast-track designation for CML EU and China: breakthrough therapy designation for CML	US: accelerated approval for Ph + CML US: full approval for Ph + CML-CP with the T315I mutation	EU and US: granted orphan drug designation for treatment of CML	–
Molecular targets	BCR-ABL (including T315I), c-KIT, FLT3, PDGFR- α, FGFR1	BCR-ABL1 Kinase	BCR-ABL1 point mutants No activity against BCR-ABL1T315I	Native BCR-ABL BCR-ABL T315I
Daily dose	40 mg every other day	80 mg 400 mg (T315I mutation)	204 mg (12–240 mg)	200–600 mg
Metabolism (CYP enzyme)	–	CYP3A4	–	–
Indications in CML	Resistance or failure to TKI	Adults with Ph + CML-CP, previously treated with ≥2 TKIs Adults with Ph + CML-CP with the T315I mutation Resistance or failure to TKI	Resistance or failure to ≥3TKIs, except for patients carrying BCR-ABL T315I mutation	Resistance or failure to ≥2 s generation TKIs
Adverse events	Thrombocytopaenia, anemia, leukopenia, neutropenia Skin pigmentation, hypocalcaemia, proteinuria, hypertriglyceridemia, arthralgia, fatigue	Skin hyperpigmentation, hypertriglyceridemia, proteinuria, thrombocytopenia Hypertension, pericardial effusion, arrythmias, retinal-vein occlusion or palpitations	Anemia, pneumonia, neutropenia, gout, thrombocytopenia, dementia, amnesia	Reversible skin toxicity; psoriasis-like skin lesions
DDI	–	Not fully characterized CYP3A4 inducers and inhibitors (e.g., imatinib) may affect asciminib level Asciminib inhibits CYP3A4, CYP2C9 and P-gp	–	–

CML-CP, chronic phase chronic myeloid leukemia; EU, European Union; FGFR1, fibroblast growth factor receptor 1; PDGFRα, platelet-derived growth factor receptor-α; P-gp, P-glycoprotein; Ph+, Philadelphia chromosome- positive; US, United States.

## Cardiovascular toxicity

### Classification and severity of cardiovascular toxicity

The types of cardiotoxicities that have been described earlier are acute, sub-chronic, early-onset chronic, and late-onset chronic cardiotoxicities. A newer classification of chemotherapy-related cardiac dysfunction (CRCD) included Type I (irreversible) and Type II (reversible) CRCD (Table 3) (1). The National Cancer Institute (NCI) developed the Common Terminology Criteria for Adverse Events (CTCAE) to ensure the consistent description and grading of cardiovascular adverse events of therapeutic agents. The severity of TKI-induced cardiovascular adverse events was also graded using NCI CTCAE (Table 4) (16, 17). Cardiovascular and pulmonary toxicities of TKIs are demonstrated in Table 5 (1–3, 5, 8, 18–21). The recent 2022 European cardio-oncology guidelines described consensus definitions for several cancer therapy-related cardiac dysfunction (CTRCD) such as heart failure and cardiomyopathy, myocarditis, cardiac arrhythmias, corrected QT interval (QTc) prolongation, vascular toxicities, and hypertension (22).

**Table 3:** Classification of cardiotoxicity.

Classic types			
Non-chronic toxicity		Chronic toxicity	
Acute cardiotoxicity	Sub-chronic cardiotoxicity	Early-onset chronic cardiotoxicity	Late-onset chronic cardiotoxicity
Rare Manifests immediately after the first drug administration, and is dose-independent Symptoms: hypotony, arrhythmias, and myocardial ischemia Usually reversible after drug discontinuation	Rare Its onset is observed in the first weeks of treatment with high doses of drugs Manifests with myocarditis or pericarditis Example: after anthracyclines administration	Develops within a few weeks after discontinuation of treatment Manifests as progressive heart failure	Develops many years after the end of the treatment and leads to heart failure
New classification			
Type I CRCD		Type II CRCD	
Irreversible Example: after anthracycline administration		Potentially reversible Induced by new-generation HER2-targeted agents and kinase inhibitors	

CRCD, chemotherapy-related cardiac dysfunction; HER2, human epidermal growth factor receptor 2.

**Table 4:** National Cancer Institute CTCAE (CTCAE 4.03) grading severity of cardiac events.

CTCAE v4.0 term	Grade 1	Grade 2	Grade 3	Grade 4	Grade 5
Heart failure	Asymptomatic with laboratory (e.g., BNP) or cardiac imaging abnormalities	Symptoms with mild to moderate activity or exertion	Severe with symptoms at rest or with minimal activity or exertion; intervention indicated	Life-threatening consequences; urgent intervention indicated (e.g., continuous IV therapy or mechanical hemodynamic support)	Death
Left ventricular systolic dysfunction	–	–	Symptomatic due to drop in ejection fraction responsive to intervention	Refractory or poorly controlled heart failure due to drop in ejection fraction; intervention such as ventricular assist device, intravenous vasopressor support, or heart transplant indicated	Death
Pulmonary edema	Radiologic findings only; minimal dyspnea on exertion	Moderate dyspnea on exertion; medical intervention indicated; limiting instrumental ADL	Severe dyspnea or dyspnea at rest; oxygen indicated; limiting self-care ADL	Life-threatening respiratory compromise; urgent intervention or intubation with ventilatory support indicated	Death
Pulmonary hypertension	Minimal dyspnea; findings on physical exam or other evaluation	Moderate dyspnea, cough; requiring evaluation by cardiac catheterization and medical intervention	Severe symptoms, associated with hypoxemia, right heart failure; oxygen indicated	Life-threatening airway consequences; urgent intervention indicated (e.g., tracheotomy or intubation)	Death
Electrocardiogram QT corrected interval prolonged^*^	QTc 450–480 ms	QTc 481–500 ms	QTc ≥501 ms on at least two separate ECGs	QTc ≥ 501 or > 60 ms change from baseline and Torsade de pointes or polymorphic ventricular tachycardia or signs/symptoms of serious arrhythmia	–
Pleural effusion	Asymptomatic; clinical or diagnostic observations only; intervention not indicated	Symptomatic; intervention indicated (e.g., diuretics or limited therapeutic thoracentesis)	Symptomatic with respiratory distress and hypoxia; surgical intervention including chest tube or pleurodesis indicated	Life-threatening respiratory or hemodynamic compromise; intubation or urgent intervention indicated	Death
Peripheral ischemia	–	Brief (<24 h) episode of ischemia managed non-surgically and without permanent deficit	Recurring or prolonged (≥24 h) and/or invasive intervention indicated	Life-threatening consequences; evidence of end organ damage; urgent operative intervention indicated	Death

*QT is the duration of ventricular depolarization and repolarization.

ADL, activities of daily living; BNP, B-Natriuretic Peptide; CTCAE, Common Terminology Criteria for Adverse Events; ECG, electrocardiogram; IV, intravenous; NCI, National Cancer Institute; QTc, corrected QT interval.

**Table 5:** Frequencies of cardiopulmonary toxicities of tyrosine kinase inhibitors in chronic myeloid leukemia.

Generation	First	Second			Third
Agents	Imatinib	Dasatinib	Nilotinib	Bosutinib	Ponatinib
QT prolongation	1–5%	5–10%	1–10%	>10%	1–5%
QT prolongation (weighted average)	3.1%	8%	2.7%	11.5%	2.5%
QTc >500 ms	0.02%	1%	0.2%	0	2.7%
Cardiomyopathy	1–2%	1.6%	<5%	–	–
Fluid retention	1–2%	1–5%	3.9%	1%	–
VAE	–	–	1.2–29.4%	–	4.9–17.1%
Pleural effusion	0–0.8%	6–35%	2%	≤5%	–
PAH	0.4%	0.45–5%	–	–	–

CML, chronic myeloid leukemia; PAD, peripheral arterial disease; PAH, pulmonary arterial hypertension; QTc, corrected QT interval; TKIs, tyrosine kinase inhibitors; VAE, vascular adverse events.

### Extent and consequences of TKI-induced cardiovascular toxicity

A case/non-case study using the Food and Drug Administration (FDA) adverse event reporting system (FEARS) database examined the cardiovascular adverse events reports of TKIs for the treatment of CML. Cases referred to reports with one or more of prespecified cardiovascular events such as cardiomyopathy, QT prolongation/torsade de pointes, pulmonary hypertension, or embolic events, whereas non-cases referred to any report for other not specified serious adverse events. FEARS allows the analysis of an enormous number of reports that helps in detecting safety signals. More than 1,300,000 adverse event reports, out of more than 20 million reports, were related to anticancer agents. Finally, 717,163 reports were analyzed after excluding non-serious events, duplicates, and aberrant cases. Cardiovascular events accounted for 64,232 cases, with 3,930 (6.1%) cases related to TKIs. In addition, TKIs were the suspected drugs in 83.2% of the cardiovascular event occurrence. Hospitalization and having fatal issues accounted for 35 and 10.1% of the cases. Of the 3,930 reports related to TKIs, 59, 21.2, 14.4, 4.4, and 1% were related to nilotinib, dasatinib, ponatinib, imatinib, and bosutinib, respectively, with 68.1% of the cases treated for CML. In comparison with other anticancer agents, cardiovascular events were more frequent with TKIs. The adjusted reporting odds ratio was 6.6 (95% confidence interval (95% CI): 5.6–7.8) for QT prolongation/torsade de pointes, 1.7 (95% CI: 1.4–2.1) for cardiac arrhythmias, 2.4 (95% CI: 2.2–2.6) for heart failure, and 3.9 (95% CI: 3.2–4.7) for pulmonary hypertension (6).

Wang et al. investigated the long-term follow-up of TKI use in CML patients (*n* = 7,119) and its association with cardiovascular toxicity using SEER (surveillance, epidemiology, and end results) program between 1992 and 2011. The program includes 18 cancer registries in the United States and covers up to approximately 28% of the American population. The authors compared patients’ outcomes in the TKI era with that of those in the pre-TKI era. CML diagnosis rate was higher in the TKI era (72.4% versus 27.6%) and was associated with significantly higher overall survival [hazard ratio (HR) 0.22; 95% CI: 0.21–0.24] than in the pre-TKI era. The probability of mortality due to cardiovascular causes was reduced in the TKI era (HR 0.72; 95% CI: 0.59–0.98) (23). Caocci et al. reported survival data of Italian patients with chronic phase CML who were on second- or third-generation TKIs (*n* = 656) and 15% of them had a history of cardiovascular disease. Following TKI therapy, 7.3 and 1.4% of patients experienced arterial occlusive events and ischemic heart disease, respectively. Peripheral arterial disease rate was higher with nilotinib and ponatinib (7.3 and 5.9%) than dasatinib and bosutinib (1.7 and 1.6%; *p* = 0.02), whereas stroke rate was higher with bosutinib and ponatinib (5 and 3%) than nilotinib and dasatinib (0.7 and 0%; *p* = 0.01), respectively. Death was reported in 37 patients of them 12 cases were related to cardiovascular complications. The 15-year overall and cardiovascular mortality free-survival rates were 83.3 and 93%, respectively (24). Interestingly, imatinib, but not dasatinib or nilotinib, caused a significant reduction in the estimated glomerular filtration rate on a long-term follow-up which may have contributed to the occurrence of ischemic events (25).

### Mechanisms of TKI-induced cardiovascular toxicity

The mechanisms of cardiovascular toxicity are generally divided into on- and off-target mechanisms. On-target mechanisms refer to the anticancer effect of TKIs through the inhibition of a molecule in the tumor cells but can also adversely affect the function of normal cells. While off-target mechanisms imply the non-selective inhibition of other kinases in normal cells, along with the kinase of the tumor cells that cause cardiotoxicity (1, 2, 7). However, the molecular mechanism of TKI-induced cardiotoxicity is not fully elucidated (1, 2, 19). Examples of target receptors that regulate signaling pathways (e.g., PI3K, MEK, and AKT) include BCR::ABL, VEGFR, epidermal growth factor receptor (EGFR), platelet-derived growth factor receptor (PDGFR), and c-KIT. TKIs are usually multi-target agents and only a few of them have one or two targets such as bosutinib (2, 3). The off-target inhibition spectrum can range from six (nilotinib) to 28 (dasatinib) target receptors. Furthermore, the effects of this inhibition depend on the specific agent and its dosage (3). There is also between-patient variability in cardiotoxicity even when treated with the same TKI, which reflects an interaction between different factors such as TKI targets, genetic predisposition, and cardiovascular risk factors (7). A new study reported a novel mechanism for the cardiovascular toxicity caused by the second- and third-generation BCR::ABL1 TKIs through their ability to activate the Rho-associated coiled-coil-containing kinase (ROCK). ROCK activity may be a prognostic biomarker for cardiovascular adverse events and, therefore, its inhibition may be promising in preventing and/or treating cardiovascular adverse events due to the implicated TKIs (26, 27).

### General approach to cardiovascular toxicity

**Figure 2:**
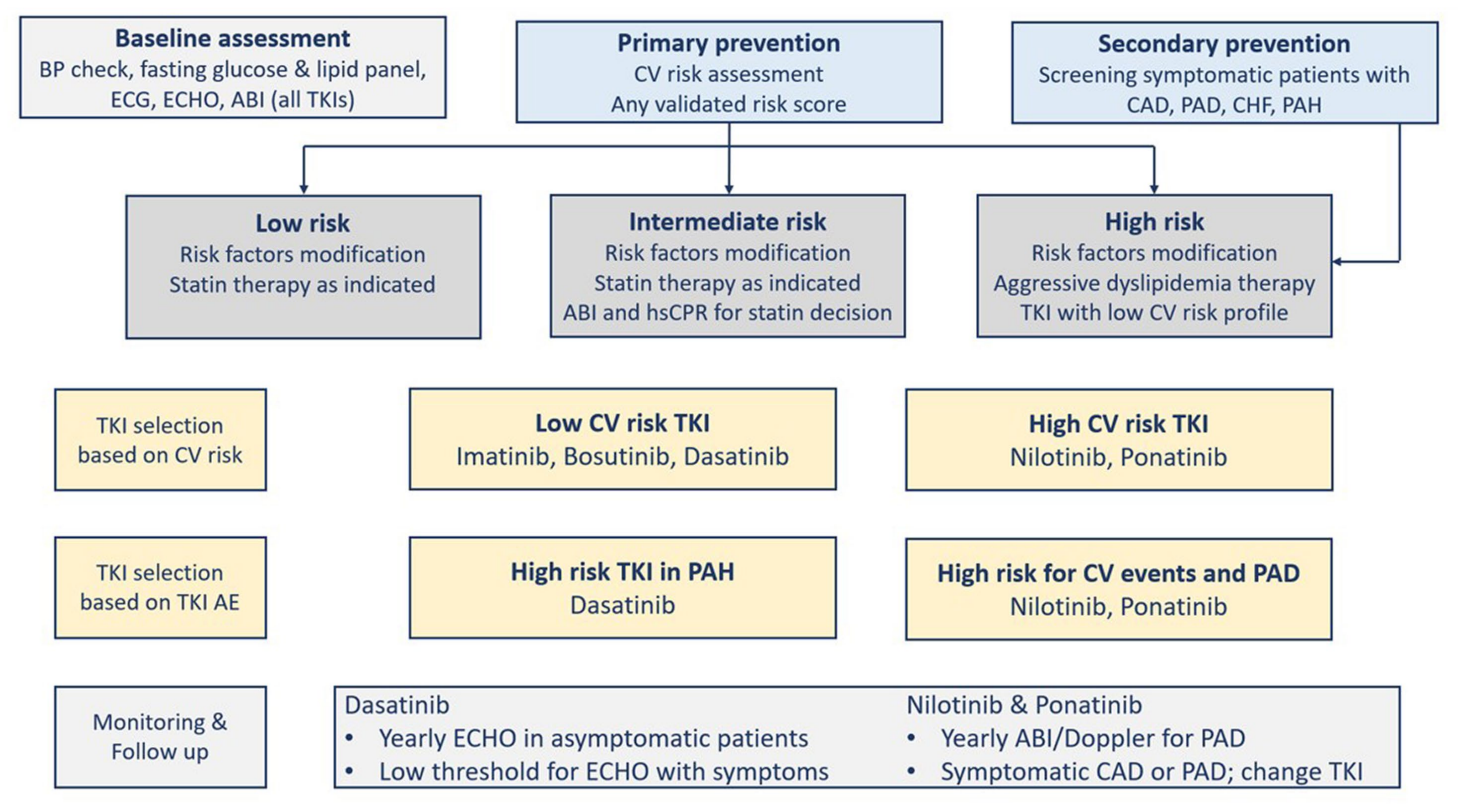
Cardiovascular risk assessment and tyrosine kinase inhibitors selection and monitoring. ABI, Ankle-brachial index; AE, Adverse Events; BP, blood pressure; CAD, coronary artery disease; CHF, congestive heart failure; CV, cardiovascular; ECG, electrocardiogram; ECHO, echocardiography; PAD, Peripheral arterial disease; PAH, pulmonary arterial hypertension; TKI, tyrosine kinase inhibitor(s).

The factors that determine the selection of a TKI comprise the goal of therapy, comorbidities, patient’s age, and the TKI’s toxicity profile (5, 28). Before deciding on the TKI therapy, cardiovascular risk factors should be evaluated. The presence of comorbidities such as coronary artery disease, hypertension, or diabetes should be closely monitored and followed up (2, 18). In addition, except for imatinib, TKIs are associated with serious cardiovascular complications (Table 1). These concerns should prompt cardiovascular risk assessment before starting TKI therapy (1, 18). Figure 2 shows the cardiovascular considerations when selecting a TKI agent (18). Given the absence of biomarkers that predict the cardiovascular risk associated with BCR::ABL1 TKI therapy, traditional risk scores derived from the general population are usually used in CML patients as well. However, such scores may underestimate the risk (27).

The prevention of cardiovascular diseases starts with the aggressive management of modifiable cardiovascular risk factors in patients with or without CML (7). The “ABCDE” approach lists the classic steps to reduce cardiovascular risk as follows: Assessment of cardiovascular disease signs and symptoms with aspirin use in select patients; Blood pressure control; Cigarette/tobacco cessation and Cholesterol level monitoring; Diabetes control and Diet/weight management; and Exercise (18, 28). International guidelines recommend calculating a risk score (e.g., Framingham, SCORE, QRISK, and Reynolds risk scores), and if the decision to consider statin is uncertain, then other factors can be considered in decision-making, for example, coronary artery calcium score, high-sensitivity C-reactive protein, or ankle-brachial index (ABI). Although such recommendations are not validated in CML patients, they can direct proper aggressive management of the modifiable risk factors. Statin therapy is effective for primary prevention in patients at high cardiovascular risk who can tolerate the therapy, particularly those on ponatinib (7). A systematic review and meta-analysis that used molecular docking to analyze the impact of aspirin and rosuvastatin on hub genes associated with nilotinib cardiotoxicity found that rosuvastatin can be effective in this case (29). Aspirin, in primary prevention, reduces the composite of serious cardiovascular events but is associated with increased gastrointestinal and intracranial bleeding risk. Thus, it needs to balance ischemic and bleeding risks. Aspirin use is more challenging in CML patients using certain TKIs (i.e., dasatinib) because it can inhibit platelet aggregation leading to increased bleeding risk (Table 6) (7). After the baseline assessment (i.e., cardiovascular risk, physical examination, vitals, laboratory tests), there should be follow-up visits at 1, 3, and 6 months (Figure 2) (18, 28). Another approach to reducing adverse events is the use of lower TKI doses and shorter durations (8). The analysis of the German CML-Study IV (Chronic Myeloid Leukemia-Study IV) has concluded that de-escalating a higher dose of imatinib (i.e., 800 mg daily) to 400 mg daily sustained deep molecular remission in 90% of patients (30).

**Table 6:** Drug–drug interactions between tyrosine kinase inhibitors and potentially interacting drugs.

	TKIs CYP inhibitor	TKIs CYP substrate	TKIs Other effects
QT prolongation (drugs with known risk and their alternatives)			
Antiarrhythmic drugs	Amiodarone, disopyramide, dofetilide, dronedarone, quinidine with imatinib DDI: moderate; monitor QT and toxicities Effect: TKI increase serum level of antiarrhythmics Mechanism: CYP3A4 inhibition	Dronedarone with bosutinib and ponatinib DDI (ponatinib): moderate; monitor QT and its risk factors DDI (bosutinib): moderate; avoid combination Effect: increase serum level of TKI Mechanism: CYP3A4 inhibition	Flecainide DDI (bosutinib): minor; no action needed DDI (dasatinib, nilotinib): moderate; monitor QT and its risk factors Amiodarone, disopyramide, dofetilide, dronedarone (only with bosutinib), procainamide, quinidine, sotalol with bosutinib, dasatinib and nilotinib DDI (bosutinib): moderate; monitor QT and its risk factors DDI (dasatinib): major; consider alternatives DDI (nilotinib): major; avoid combination Effect: enhance QTc-prolonging effect of TKI Mechanism: additive effect
Alternatives	–		
Common antimicrobial drugs	–	Clarithromycin (major), fluconazole (moderate) with bosutinib DDI: moderate; avoid combination DDI: major; avoid combination Clarithromycin (moderate), fluconazole (minor) with imatinib Clarithromycin (major), fluconazole (moderate) with ponatinib Fluconazole (moderate) with dasatinib DDI: minor; no action needed DDI: moderate; monitor TKI toxicities DDI: major; consider alternatives Effect: increase serum level of TKI Mechanism: CYP3A4 inhibition	Clarithromycin (major), azithromycin (dasatinib), fluconazole (nilotinib), levofloxacin, moxifloxacin, pentamidine (IV) (moderate), ciprofloxacin (minor) with nilotinib and dasatinib Azithromycin, levofloxacin, moxifloxacin, Pentamidine with bosutinib (minor) DDI: minor; no action needed DDI: moderate; monitor QT and its risk factors DDI: major; consider alternatives Effect: enhance QTc-prolonging effect Mechanism: additive effect
Alternatives	Penicillin, cephalosporins, doxycycline, anidulafungin		
Prokinetic and antiemetic drugs	Droperidol (moderate) with nilotinib DDI: moderate; monitor for QT and its risk factors Domperidone (major) with imatinib and nilotinib DDI: major; avoid combination Effect: increase serum level of antiemetics Mechanism: CYP3A4 inhibition	–	Ondansetron (moderate) with nilotinib Chlorpromazine (moderate), domperidone, droperidol (minor) with bosutinib DDI: minor; no action needed DDI: moderate; monitor QT and its risk factors Chlorpromazine with dasatinib and nilotinib DDI (dasatinib): major; consider alternatives DDI (nilotinib): major; avoid combination Effect: enhance QTc-prolonging effect Mechanism: additive effect Domperidone, droperidol with dasatinib DDI (droperidol): moderate; monitor QT and its risk factors DDI (domperidone): moderate; consider alternatives Effect: enhance QTc-prolonging effect Mechanism: additive effect
Metoclopramide (conditional risk)	–	–	Metoclopramide (minor) with dasatinib and nilotinib DDI: minor; no action needed Effect: enhance QTc-prolonging effect Mechanism: additive effect
Alternatives: Aprepitant, fosaprepitant, palonosetron	Aprepitant (not with ponatinib), fosaprepitant with imatinib, nilotinib, and ponatinib DDI: moderate; avoid combination Effect: increase serum level of antiemetics Mechanism: CYP3A4 inhibition	Aprepitant with bosutinib and dasatinib DDI (dasatinib): moderate; monitor TKI toxicities DDI (bosutinib): moderate; avoid combination Effect: increase serum level of TKI Mechanism: CYP3A4 inhibition	–
Antipsychotic drugs	Haloperidol, thioridazine with imatinib Pimozide with ponatinib DDI (haloperidol): minor; no action needed DDI (thioridazine): moderate; monitor for thioridazine toxicities DDI (pimozide): moderate; avoid combination Effect: increase serum level of antipsychotics Mechanism: CYP3A4 (haloperidol/pimozide) or CYP2D6 (thioridazine) inhibition	–	Pimozide, thioridazine with bosutinib (minor) Pimozide with nilotinib Pimozide, thioridazine with dasatinib DDI: minor; no action needed DDI (thioridazine): moderate; monitor QT and its risk factors DDI (pimozide): moderate; avoid combination Effect: enhance QTc-prolonging effect Mechanism: additive effect Haloperidol with dasatinib, bosutinib, and nilotinib Thioridazine with nilotinib DDI: moderate; monitor QT and its risk factors Effect: enhance QTc-prolonging effect Mechanism: additive effect
Alternatives	Brexpiprazole (moderate) with ponatinib and nilotinib DDI: moderate; monitor Effect: increase serum level of brexpiprazole Mechanism: CYP3A4 inhibition		
Antidepressants	–	–	Citalopram and escitalopram with bosutinib, dasatinib and nilotinib Effect: enhance QTc-prolonging effect of TKI DDI (bosutinib): minor; no action needed DDI (dasatinib/nilotinib): moderate; monitor QT and its risk factors Mechanism: additive effect
Alternatives: Desvenlafaxine, bupropion, vortioxetine	–	–	Desvenlafaxine, vortioxetine with dasatinib DDI: major; monitor bleeding Effect: enhance antiplatelet properties Mechanism: additive effect
Pulmonary arterial hypertension			
Endothelin receptor antagonists	Macitentan with imatinib, nilotinib DDI: moderate; monitor for macitentan toxicities Effect: increase serum level of macitentan Mechanism: CYP3A4 inhibition	Bosentan with imatinib, dasatinib, nilotinib, ponatinib, and bosutinib DDI: moderate; monitor TKI efficacy DDI (Bosutinib): major; avoid combination Effect: decrease serum level of TKI Mechanism: CYP3A4 induction	–
Phosphodiesterase type-5 inhibitors	Sildenafil/Tadalafil with imatinib, and nilotinib DDI: moderate; monitor for adverse effects (e.g., hypotension, headache) Effect: increase serum level of sildenafil Mechanism: CYP3A4 inhibition	–	–
Other classes	Soluble cyclic cGMP stimulators, prostacyclin receptor agonists, and prostacyclin derivatives do not interact with TKIs		
Primary and secondary cardiovascular disease prevention			
Antiplatelets	Ticagrelor with imatinib and nilotinib DDI: moderate; monitor for adverse effects (e.g., bleeding) Effect: increase serum level of ticagrelor Mechanism: CYP3A4 inhibition	–	Aspirin, clopidogrel, prasugrel, ticagrelor with dasatinib DDI: major; monitor for bleeding Effect: enhance antiplatelet properties Mechanism: additive effect; dasatinib carries risk of thrombocytopenia
Statin*	Atorvastatin, lovastatin, simvastatin with imatinib and nilotinib DDI: moderate; monitor for increased statin adverse effects (e.g., myopathy) Effect: increase serum level of statin Mechanism: CYP3A4 inhibition	–	–
Heart failure			
RAAS^$^	Eplerenone with imatinib and nilotinib DDI: moderate; use maximum of 25 mg daily for heart failure and 25 mg twice for HTN Effect: increase serum level of eplerenone Mechanism: CYP3A4 inhibition	–	–
Other classes	Beta-blockers except for sotalol (see antiarrhythmics), thiazide or loop diuretics, and SGLT2i do not interact with TKIs		
Other relevant drug classes			
Fibrates	–	–	Gemfibrozil with imatinib DDI: moderate; monitor therapy Effect: decrease serum level of imatinib Mechanism: possible inhibition of imatinib intestinal absorption and conversion to active metabolite (CYP1C8)
DHP-CCB	Amlodipine (minor), felodipine (moderate), nifedipine (moderate) DDI: monitor for adverse effects (e.g., edema, hypotension) Effect: increase serum level of DHP-CCB Mechanism: CYP3A4 inhibition	–	–
Non-DHP-CCB (as CYP3A4 inhibitors)	–	Diltiazem/Verapamil with bosutinib/ponatinib (moderate) and nilotinib (minor) DDI (bosutinib): moderate; avoid combination DDI (ponatinib/nilotinib): monitor TKI toxicities Effect: increase serum level of TKI Mechanism: CYP3A4 inhibition	–
Non-DHP-CCB (as substrates)	Diltiazem (minor)/Verapamil (moderate) with imatinib and nilotinib DDI: monitor for side effects (e.g., hypotension, bradycardia) Effect: increase serum level of non-DHP CCB Mechanism: CYP3A4 inhibition	–	–

ACEI, angiotensin-converting enzyme inhibitors; ARB, angiotensin receptor blockers; ARNI, angiotensin receptor and neprilysin inhibitors; CCB, calcium channel blockers; cGMP, cyclic guanosine monophosphate; DHP, dihydropyridine; DDI, drug–drug interaction; HTN, hypertension; IV, intravenous; PCSK9i, proprotein convertase subtilisin/kexin type 9; QTc, corrected QT; RAAS, renin-angiotensin-aldosterone system; SGLT2i, sodium-glucose cotransporter-2 inhibitors; TKI(s), tyrosine kinase inhibitor(s).

*No DDI with ezetimibe, PCSK9i, or other unmentioned statins.

^$^No DDI with ACEI/ARB/ARNI, or spironolactone.

Reference: Lexicomp^®^ available from: http://online.lexi.com/lco/action/interact; accessed on December 8, 2022.

### Vascular adverse events

#### Occurrence and consequences

TKI-associated vascular events usually involve arterial beds (i.e., cerebral, coronary, and peripheral), which generally arise from various etiologies, for example, atherosclerosis, vasospasm, thrombosis, vasospasm, or vasculitis. Unfortunately, the definition of TKI-associated vascular events has not been consistent among the TKI studies and the vascular events have not been adjudicated in CML studies. A study, for example, defined vascular adverse events as myocardial infarction, ischemic stroke, venous thromboembolism, and arterial occlusive disease (31). The most frequently used oncology-related terminology was peripheral artery (or arterial) occlusive disease (PAOD) (18). PAOD refers to the obstruction or occlusion of large arteries (but not coronary arteries), aortic arch, or the arteries supplying the central nervous system. PAOD shares risk factors similar to those contributing to atherosclerosis such as age, obesity, smoking, male gender, dyslipidemia, diabetes, and hypertension. Kim et al. in their study defined a clinically manifest PAOD as experiencing an acute PAOD event or having typical peripheral ulcerations or imaging-identified lesions. PAOD diagnosis can be established by abnormal ABI measurement (i.e., value <0.9) and the use of imaging modalities such as angiography or ultrasonography can aid in identifying the affected arteries and localizing the related lesions (32). Measuring the ABI in CML patients verified the associated proatherogenic effects, as abnormal ABI is sensitive and specific in detecting peripheral artery disease (8). As such, the mechanism that explains the effect of TKI therapy may be the accumulation of atherosclerosis in numerous arterial beds (18). In a prospective screening for PAOD in chronic phase CML patients on TKIs, ABI was available for 81% of patients and 18.6% of them had pathological or abnormal ABI. The rates of pathological ABI were significantly higher with nilotinib use, either as first (26%; *p* = 0.0297) or second-line (35.7%; *p* = 0.0029) therapy than with imatinib first-line therapy (6.3%), accounting for PAOD relative risk of 10.3 with nilotinib first-line therapy. Clinical manifestations of PAOD were only seen in patients on nilotinib. In the first-line nilotinib group, pathological ABI developed after 21–56 months of therapy (32).

Vascular events are clinically important adverse events that were observed with second and third generations TKIs. Earlier, there was little data about the incidence of vascular adverse events with long-term TKI use and the predisposing factors because initial Phase I and II trials did not capture these events (8, 33). However, their frequencies considerably varied in the subsequent reports (e.g., 1–29% over 2 years) (8). Dahlén et al. retrospectively detected vascular events incidence in Swedish CML patients (*n* = 896) using first- and second-generation TKI therapy with a median follow-up of 4.2 years. The relative risks for arterial and venous events were 1.5 (95% CI: 1.1–2.1) and 2.0 (95% CI: 1.2–3.3), respectively. Myocardial infarction rates were higher with nilotinib or dasatinib than with imatinib use. However, the authors stated that patients may have been prescribed multiple TKI agents during the study (34). The retrospective evidence showed that the vascular events rate (1%) was significantly lower with imatinib than that with nilotinib or ponatinib use. Similarly, the events rate was low with both dasatinib and bosutinib (8). Chen et al. tested the incidence of vascular adverse events in Taiwanese CML patients (*n* = 1,111) treated with dasatinib, imatinib, or nilotinib and showed that the latter TKI significantly increased the incidence rate compared with imatinib (HR 3.13; 95% CI: 1.30–7.51), while dasatinib was associated with a non-significant numerical rise in events rate (HR 1.71; 95% CI: 0.71–4.26). The identified risk factors included old age, nilotinib use, and previous history of cerebrovascular disease (31). In a meta-analysis of randomized trials, the use of dasatinib [odds ratio (OR) 3.86, 95% CI: 1.33–11.18], nilotinib (OR 3.42, 95% CI: 2.07–5.63), and ponatinib (OR 3.47, 95% CI: 1.23–9.78) was associated with higher vascular occlusive events rate than with imatinib. Only a trend of an increased risk was found with bosutinib use (OR 2.77, 95% CI: 0.39–19.77) (33).

The reported rate of PAOD related to nilotinib ranged from 1.2 to 12.5% (32). Cardiovascular events associated with nilotinib seem to appear at a long-term follow-up (18). For example, at the 12- and 24-month follow-up of the ENESTnd (Evaluating Nilotinib Efficacy and Safety in Clinical Trials–Newly Diagnosed Patients) trial, there was not a safety signal of vascular adverse events (35, 36). Subsequently, PAOD started to be apparent after a minimum of three-year follow-up and did not necessitate drug discontinuation. Most of the patients with PAOD events had baseline risk factors for PAOD and more patients treated with nilotinib experienced ischemic heart adverse events (37). Similar pattern was seen at the 5-year follow-up (38). At a 10-year follow-up, the cumulative cardiovascular events rates continued to increase with nilotinib use for up to 16.5% (300 mg twice daily) and 23.5% (400 mg twice daily) compared with 3.6% for imatinib, even in patients with low Framingham risk score (39). In the ENESTnd extension study, dose escalation from 300 mg to 400 mg twice daily resulted in only a few new adverse episodes (40). The mechanism of vascular adverse events of nilotinib is multifactorial. The direct effect of nilotinib on the vascular cells by exerting proatherogenic and antiangiogenic effects leads to arterial stenosis and inhibition of repair mechanisms, respectively. Other nilotinib-related factors comprise vasospasms and metabolic effects (i.e., elevated cholesterol and glucose levels), all of which can provoke atherosclerosis (8, 18, 33). Ponatinib caused vascular adverse events even in patients without prior treatment with nilotinib, which led to ponatinib transient withdrawal from the market by the FDA in 2013 (8, 18). There is no sufficient data available on the vascular effects of ponatinib, but it may hinder the growth and survival of endothelial cells. Risk factors for TKI-related adverse events comprise those implicated in the process of atherosclerosis such as age, smoking, obesity, diabetes, hypertension, and hypercholesterolemia (8, 18, 33). In a prospective study, the rate of adverse events with ponatinib use was 8.9 and 17.1% after 11 and 24 months, respectively (8).

#### Management

Preventing vascular adverse events is of paramount importance in the management of CML patients because their prognosis is considered good when treated with TKIs, but vascular adverse events can affect their morbidity and transplant eligibility. Risk factors for PAOD are common in the general population which necessitates individual risk determination using a validated risk score along with aggressive treatment of the underlying comorbidities and modifiable risk factors as discussed above (Figure 2). Individual risk scores can also help in selecting a safe and optimal TKI in CML patients, which is an important initial step in the prevention of adverse events. In patients with multiple risk factors or high-risk scores, nilotinib or ponatinib are not the preferred TKIs if other agents can be considered (8, 33). It was suggested to reserve ponatinib for advanced disease in the presence of a T315I mutation (33). Moreover, the presence of multiple risk factors for PAOD subjects the patient to pleural effusion while on dasatinib. Thus, the aforementioned agents (dasatinib, nilotinib, and ponatinib) are not the optimal choice, particularly in the elderly (8). The claim that bosutinib is a better option should be confirmed by clinical trials. However, the management of vascular adverse events that develop during nilotinib or ponatinib therapy should be individualized. Patients with low-grade PAOD, i.e., grades I and II, should be treated with optimal PAOD therapy and aggressive management of all cardiovascular risk factors, whereas the management of patients with high-grade PAOD, i.e., grades III and IV is more difficult, and some patients need to continue nilotinib or ponatinib based on the mutation status of the CML. Otherwise, the TKI should be stopped or replaced by another agent. Higher doses of TKIs are associated with more clinically relevant vascular adverse events. As a result, lower TKI doses and shorter durations of therapy may reduce the adverse event rates (8). Dorer et al. pooled data from three studies (*n* = 671) and found a significant correlation between the intensity of ponatinib dose and arterial occlusive events risk (OR 1.71). Ponatinib dose reduction by 15 mg daily decreased arterial occlusive events risk by 33% (41). The OPTIC (Optimizing Ponatinib Treatment in CP-CML) Phase II study explored a dose-reduction approach to examine three ponatinib starting doses (45, 30, and 15 mg daily) on the safety and efficacy in highly resistant CML patients. For 45-mg and 30-mg groups, the dose was reduced to 15 mg daily when treatment response was seen (i.e., BCR::ABL1IS transcript level is 1% or less). The response at 12 months (i.e., primary endpoint) was attained in 44.1, 29, and 23.1%, respectively. The arterial occlusive events of severity grade 3 or above in response to treatment occurred in three patients in the 15-mg group and five patients in each of the other groups. The investigators concluded that optimal efficacy and safety outcome was attained when starting treatment with 45 mg daily and then reducing it to 15 mg daily upon achieving a response (42). Patients with PAOD who require interventional revascularization due to cerebral ischemia or myocardial infarction should consider switching to another TKI therapy, along with the guidelines-recommended antiplatelet and anticoagulation therapies (8).

### QT prolongation

#### Occurrence and consequences

QT prolongation and torsade de pointes are considered acute events. Their incidence was higher with TKIs (6.8%) than that with other anticancer agents (1.2%). The occurrence of QT prolongation was associated with increased polymorphic ventricular arrhythmia and the consequent fatal arrythmias and sudden cardiac death (2). Although the absolute risk is minimal, the risk of torsade de pointes has been associated with prolonged QT intervals, especially when QTc is above 500 milliseconds (ms) (16). There is a 2–3-fold increased risk for torsade de pointes when QTc is 500 ms or above compared with a value of less than 500 ms (22). When quantifying the risk, studies found that each 10-ms increase in QTc interval causes an increase of approximately 5–7% in cardiac events risks such as syncope, cardiac arrest, or death (19). Third-generation TKIs have been associated with QT prolongation (1). QT prolongation warranted a black box warning for nilotinib (18, 19). One of the on-target proposed mechanisms for QT prolongation is the inhibition of the PI3K signaling pathway that led to changes in sodium and potassium current, accounting for more than 70% of the overall prolongation (1, 2, 16). An off-target mechanism occurs through the interference of TKIs with cardiomyocytes potassium channel protein called human Ether-a-go-go (hERG) (2, 19). hERG facilitates ventricular repolarization of potassium current potential during phases 2 and 3 of the action potential. TKIs have variable effects on QT prolongation, with dasatinib being the most implicated agent (2). Potential risk factors that can potentiate the risk of QT prolongation include drug–drug interaction with inhibitors of cytochrome P450 (CYP) 3A4 (CYP3A4) and CYP2D6 enzymes (Table 6), genetic predisposition, electrolyte disturbances, and conditions (e.g., renal or hepatic failure) that reduce the elimination of TKIs (16, 19). Overall, the rate of drug-induced QT prolongation is considered low and patients with malignancies, in comparison with healthy individuals, may be at higher risk for torsade de pointes due to their underlying disease and its treatment. In addition, those with advanced disease may accept the risk of cardiotoxicities of their therapy that prolongs their survival unlike patients with less-advanced cancers who may not (16).

#### Management

The QT interval is usually measured based on leads II and V5 because they show the earliest onset of the QRS complex and the T-wave offset. There are different formulae to calculate the QT interval which correct the QT interval for the heart rate. The most common ones are the Bazett, Fridericia, and Hodges formulas (19). Although none of them is superior to another (1), the Fridericia formula is the one recommended in cancer patients and compared with other formulas, it has shown fewer errors at high and low heart rates (43). In the general population, the upper 99% limits of normal QTc values are 450 ms for men and 460 ms for women. QTc prolongation to 480 ms or above during cancer therapy is relatively frequent and requires closer monitoring. The change in QT interval of more than 60 ms from baseline does not necessitate an alteration in treatment decision as long as QTc remains below 500 ms (22). There is no risk score to help identify the patient’s risk factors for QTc prolongation while on TKI therapy (19). However, Tisdale et al. developed and validated a risk score for the prediction of QT prolongation in hospitalized patients which may guide monitoring and therapeutic decisions. The risk score classifies patients into three risk categories as low (score < 7), moderate (score 7–10), and high (score ≥ 11), according to a calculated score based on the presence of nine risk factors (age ≥ 68 years, female sex, loop diuretic use, serum potassium ≤3.5 mEq/L, admission QTc ≥ 450 ms, QTc-prolonging drugs, acute myocardial infarction, sepsis, and heart failure) (44).

**Figure 3:**
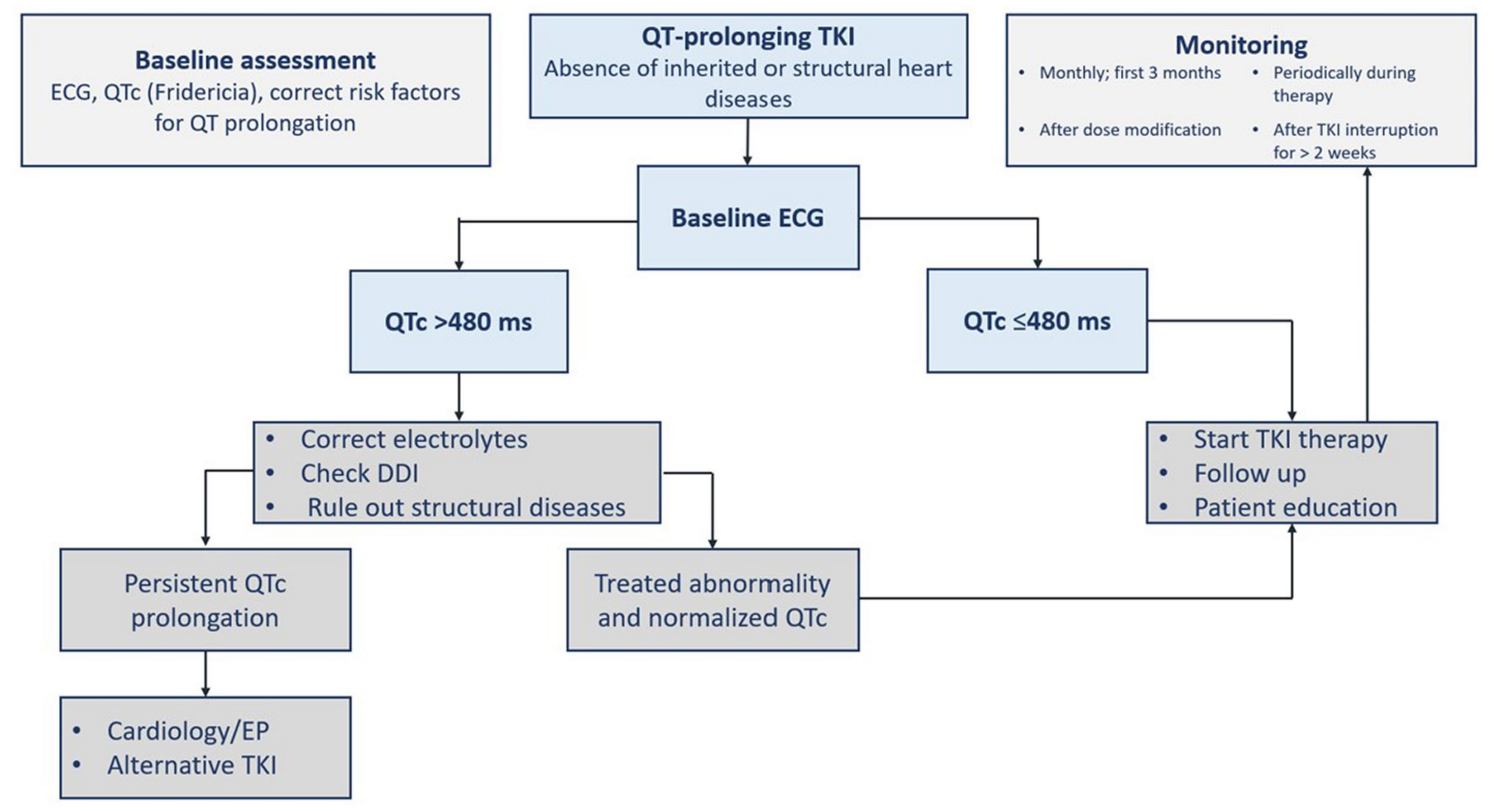
Assessment of QTc prolongation before and during tyrosine kinase inhibitor therapy. ECG, electrocardiogram; DDI, drug-drug interaction; EP, electrophysiology; TKI, tyrosine kinase inhibitor(s); QTc, corrected QT interval.

Before receiving a QT prolonging-TKI, a complete past medical and medication history should be collected (2, 16, 19). There are different reported congenital (e.g., Brugada and congenital long QT syndromes) and acquired reasons for QT prolongation. Acquired causes can be cardiac (e.g., cardiomyopathy and ischemia), metabolic (e.g., hypothyroidism and electrolytes), drug-induced (e.g., antiarrhythmics, psychotropics, and antimicrobials), or others (e.g., hypothermia) (16). Baseline and regular electrocardiogram (ECG) with QTc monitoring are advised during TKI therapy. Other monitoring measurements include blood pressure, electrolytes, thyroid hormones, B-type natriuretic peptide (BNP), and cardiac biomarkers (1, 2, 16). Immediate and careful patient evaluation should be performed upon detecting a prolonged QTc interval as defined (19). General measures comprise correcting and monitoring identifiable causes such as electrolytes imbalance (i.e., hypokalemia, hypomagnesemia, and hypocalcemia) (2, 16), and the presence of QT-prolonging drugs that interact with TKIs. The drug regimen should be modified or discontinued (Table 6). The presence of palpitation, syncope or presyncope, or QT prolongation with new-onset bradycardia of a rate less than 60 beats per minute and high-degree heart block should prompt immediate evaluation with continuous monitoring. The ECG must be repeated on a daily basis until QT prolongation is resolved. Correcting the QT prolongation by using drugs should be considered if there are concerning ECG signs for torsade de pointes. Treatment includes intravenous magnesium sulfate, beta-adrenergic agent (e.g., isoproterenol), lidocaine infusion and temporary pacing for refractory cases, and changing rate setting in patients with implantable cardiac devices (19). A proposed management algorithm for QT interval prolongation surveillance before and during TKI therapy is summarized in Figure 3 (2, 16, 18, 19).

### Heart failure

#### Occurrence and consequences

Cardiomyopathy or cardiac dysfunction induced by chemotherapy may range from asymptomatic myocardial injury [i.e., evident by a rise in cardiac troponin or decrease in global longitudinal strain (GLS)] to severe heart failure with reduced left ventricular ejection fraction (LVEF). Chemotherapy-induced cardiomyopathy is usually defined as a reduction in LVEF by more than 10% points and reaching a value below the lower limit of normal. Among the other cardiomyopathy types, it has the worse prognosis, adding to that the need for chemotherapy interruption or switching to a less effective alternative, hence leading to reduced survival in cancer patients (31, 45). Heart failure due to cardiomyopathy is a serious adverse event of TKI treatment. Dasatinib caused serious cardiotoxicity including heart failure and cardiomyopathy in 1.6% of patients receiving it. Mitochondrial damage, cardiac energy balance alterations, and contractile protein dysfunction can lead to left ventricular systolic dysfunction and consequent heart failure. It was hypothesized that inhibiting PDGFR and other tyrosine kinase receptors affects the myocytes’ normal response to hypertensive stress. Cardiac damage may result from interfering with the ribosomal S6 kinase family as it inhibits the phosphorylation of apoptosis-activating factors and thus determines the survival of the cardiomyocytes (1).

#### Management

**Figure 4:**
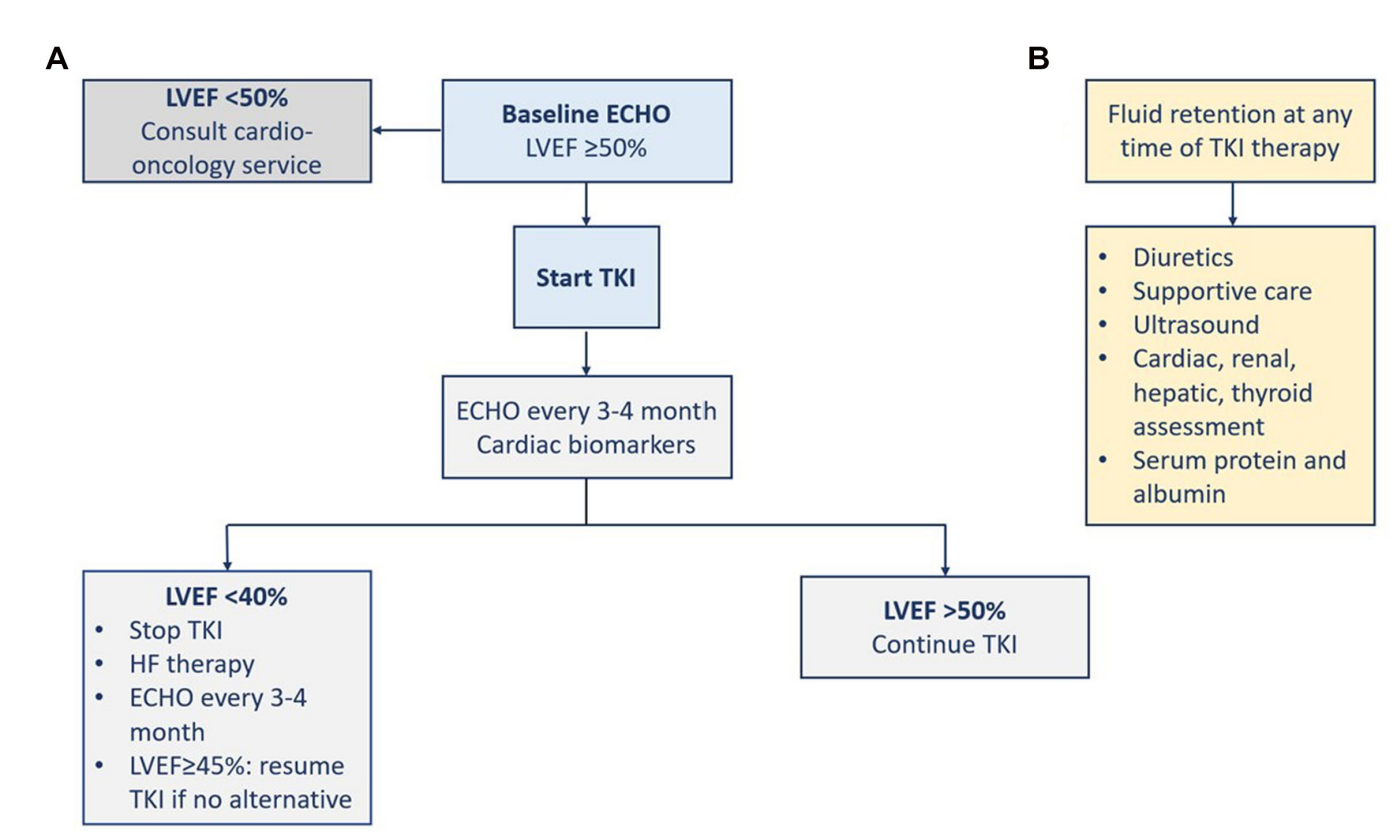
Management of heart failure **(A)** and fluid retention **(B)**. ECHO, echocardiography; LVEF, left ventricular ejection fraction; TKI, tyrosine kinase inhibitor.

The risk of systolic dysfunction and heart failure is probably underestimated in the relevant studies (1). TKI-induced cardiotoxicity occurs independently of the dose and at any time throughout therapy (i.e., a few days to months). Early recognition of cardiomyopathy and initiation of heart failure therapy increases the chances of left ventricular function improvement (45). Patients on potentially cardiotoxic agents should be closely monitored for any cardiac dysfunction. Monitoring of patients on a TKI with unclear risk such as imatinib is only recommended in the presence of predisposing comorbidities, or signs and symptoms of cardiac disease. Patients with cancer therapy-induced heart failure should be managed according to the international heart failure guidelines regardless of their cancer status. Before the initiation of TKI therapy, LVEF should be assessed at baseline (1, 45). For patients on TKI, the surveillance strategy is not supported by adequate evidence. The gold standard in chemotherapy-induced cardiomyopathy monitoring is the assessment of LVEF. The utilization of cardiac troponin and BNP has been proposed and supported by evidence for the early detection of subclinical cardiotoxicity (45). For asymptomatic heart failure, the European cardio-oncology guidelines defined the respective CTRCD based on reductions in LVEF and/or changes in GLS (22). Thavendiranathan et al. in their study support the utilization of GLS to guide cardioprotective therapy to prevent CTRCD and reductions in LVEF (46). Earlier, Negishi et al. found that GLS was an early predictor of reductions in LVEF as a consequence of cancer therapy (47). Since chemotherapy-induced heart failure usually improves with guidelines-directed medical therapy, implantable cardiac devices should be reserved for patients with persistent systolic dysfunction or when there is a prognostic mortality benefit. In the absence of dedicated randomized trials, evidence from clinical practice showed a potential long-term efficacy of CML treatment. Patient education about the potential risk, the possibility of prevention, and the management approaches can help benefit from long-term therapy with TKIs (Figure 4) (16).

### Fluid retention

#### Occurrence and management

Fluid retention induced by some TKIs can result in pericardial effusion or pulmonary edema (1). At the 5-year follow-up of the ENESTnd trial, severe fluid retention was more frequent with imatinib (23.2%) than with the two doses of nilotinib (11.1 and 14.4%). Severe fluid retention included peripheral edema, pleural effusion, pulmonary edema, pericardial effusion, or cardiac tamponade (38). However, at the 10-year follow-up, severe fluid retention was infrequent (39). Although it has been described with the use of imatinib or bosutinib, heart failure was rarely observed (~1%). Pulmonary edema due to fluid retention was observed with dasatinib use. The inhibition of PDGFR signaling was proposed as a possible mechanism since it regulates the homeostasis of interstitial fluid. The standard management of fluid retention is diuretics and supportive care. The dose of the TKI can be reduced or the TKI can be stopped if needed. There should be an assessment for the cardiac, renal, hepatic, and thyroid functions, in addition to the serum levels of protein and albumin. Ultrasound modality is indicated to diagnose and monitor patients with any sign of pulmonary edema, pericardial or pleural effusion, and ascites (Figure 4) (1).

## Pulmonary toxicity

The incidence of TKI-induced pulmonary toxicities is less than 1%. The frequent toxicities included pleural effusion, pulmonary arterial hypertension (PAH), pulmonary edema, interstitial lung disease, pneumonitis, chylothorax, and upper respiratory tract infection (5). The available data about the mechanisms of pulmonary toxicity involves that of dasatinib. The long-term toxicity data for both bosutinib and ponatinib is scarcely given their relatively recent approval. Luckily, the adverse events are usually reversible after drug discontinuation. Although there are various possible overlapping mechanisms for TKI-induced pulmonary toxicities, the strongly implicated ones are, direct cell injury, endothelial cell dysfunction, excess reactive oxygen species at cellular levels, or indirect inflammatory-mediated events. The management of TKI-induced pulmonary toxicity should be individualized considering the severity of respiratory status and hematology service involvement (3).

### Pleural effusions and interstitial lung diseases

#### Occurrence and consequences

The incidence of pleural effusions differs between the TKIs, with dasatinib being the most implicated TKI. Interstitial lung diseases are rarer in occurrence but both conditions should be suspected in TKI-treated patients when experiencing non-specific respiratory symptoms. None of the conditions was reported with ponatinib. An incidence of pleural effusion of less than 5% occurred with bosutinib over 5 years, with more frequent rates in older patients (3). Chylothorax, a subgroup of pleural effusion, is a rare pulmonary toxicity that was reported with dasatinib (5). A retrospective study (*n* = 212) found that 55% of chronic phase CML patients on dasatinib had adverse events. Pleural effusions were the most frequent ones (25%) and the predominant reason for permanent drug discontinuation. The authors suggested the use of the lowest effective dose particularly in older patients to decrease the risk of pleura effusion occurrence (34, 48). The FDA has announced a warning about the cardiopulmonary risk of dasatinib therapy (18). However, the response to dasatinib therapy and the survival (i.e., both progression-free and overall) did not differ between patients who developed pleural effusion and who did not (3). The significant risk factors for the development of pleural effusion associated with dasatinib included twice daily dasatinib schedule, duration of CML, skin rash, duration of dasatinib therapy, and comorbidities such as cardiac history, hypertension, hypercholesterolemia, and autoimmune disease (3, 5, 8).

#### Management

A detailed investigation for pleural effusion is warranted given the various possible causes of it. If there is a sufficient effusion volume, diagnostic thoracentesis is indicated to exclude pleural infection and to eliminate other differential diagnoses. The use of diuretics and corticosteroids has been described in the literature but has not been tested in trials. Their use may be dependent on the causative element of the pleural effusion. The size and consequences of the effusion usually dictate specific management. Close clinical and diagnostic monitoring can be sufficient with minimal effusion volume, whereas effusions with moderate or large volumes may necessitate reducing the TKI dose, withdrawing, or changing the used TKI. Since the occurrence of interstitial lung diseases with TKI therapy is rare, other possible causes should be ruled out. TKI-induced pneumonitis resolves spontaneously with TKI discontinuation or corticosteroid use. It has been reported that interstitial lung disease due to imatinib did not occur when changing to nilotinib. However, re-challenging with the use of the offending TKI can be considered based on the risk–benefit assessment for an individual patient (3).

Dasatinib is associated with high occurrence rates of pleural effusion, with the patient’s age being the most significant factor for its incidence. However, it is usually a reversible adverse event in most cases (5). Dose reduction (e.g., 100 mg once daily), dose interruption, TKI discontinuation, and drug therapy have been suggested for the management of dasatinib-induced pleural effusion (3, 5, 7). Naqvi et al. updated the results of their initial study and continued to demonstrate the efficacy and tolerability of dasatinib 50 gm daily for newly diagnosed CML patients in the chronic phase (49, 50), with 6% of patients experiencing pleural effusion and 80% of them needed further dose reduction (50). In the multicenter single-arm DAVLEC (DAsatinib, Very Low-dose, for Elderly CML-CP patients) Phase II Japanese study (*n* = 52), the standard dasatinib starting dose was reduced by 20% and 20 mg daily was administered in new diagnosed elderly patients above 70 years of age. Pleural effusion, not pulmonary hypertension, was observed in 7.7% of patients. The investigators encouraged dose reduction consideration and conducting more studies on larger and more diverse cohorts (51). However, dasatinib dose reduction did not prevent the recurrence of pleural effusion in all the cases. Drug withdrawal was reported in 22% of patients who developed dasatinib-induced pleural effusion without the need for a therapeutic thoracentesis. Switching from dasatinib to bosutinib resulted in the recurrence of pleural effusion in 30% of the cases. Although not widely used, therapeutic drug monitoring of TKIs may minimize pleural effusion rates (3). There are some reports on the use of diuretics and corticosteroids as mentioned above (3, 7). The use of vasopressin V_2_-antagonist (tolvaptan) with diuretics, in some reports, improved pleural effusion and allowed continuing or reintroducing dasatinib (3).

**Figure 5:**
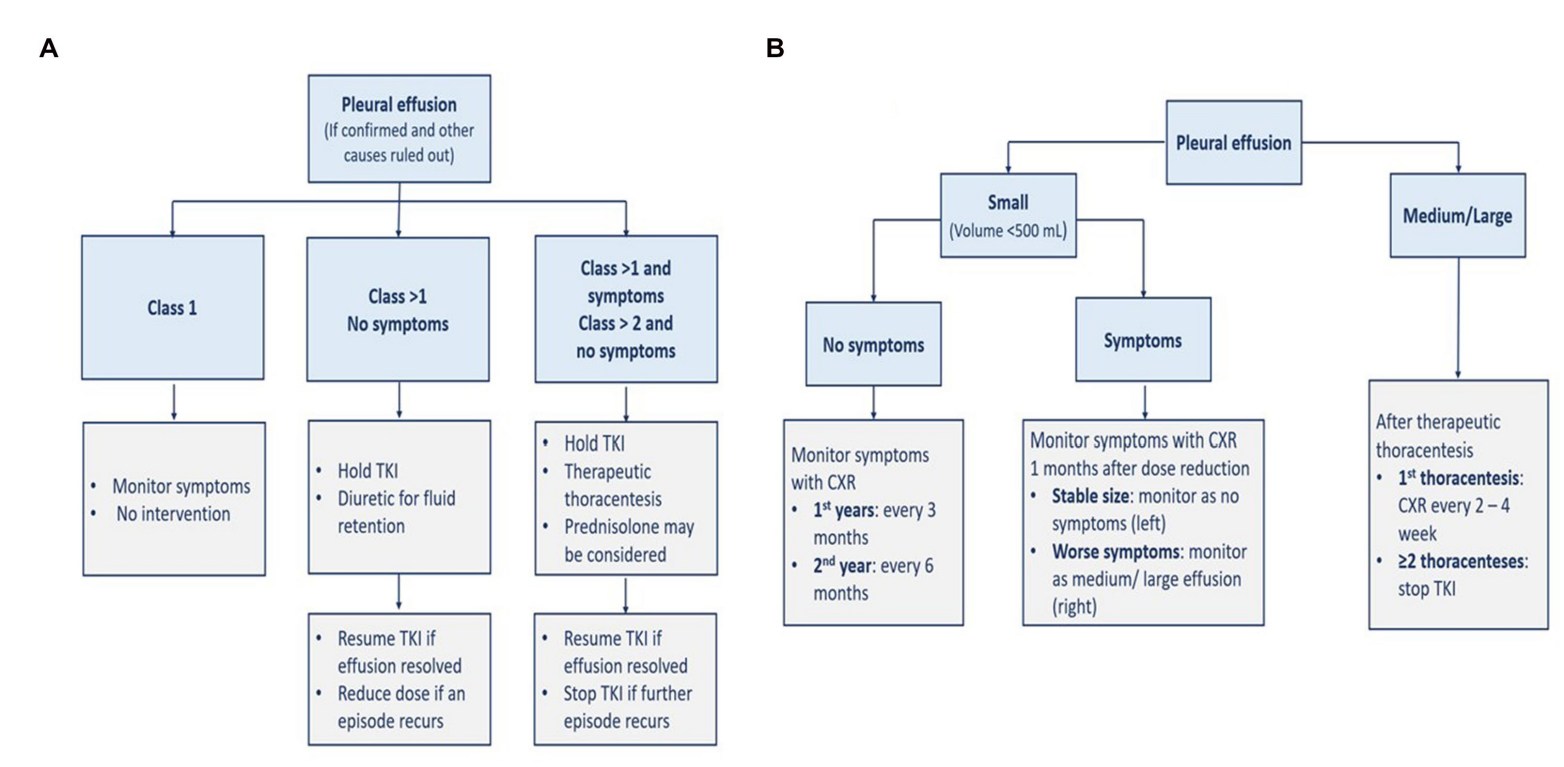
Management **(A)** and monitoring **(B)** of pleural effusion. CXR, chest x-ray; TKI, tyrosine kinase inhibitor.

Patients with Class 1 pleural effusion do not require intervention. In asymptomatic patients with Class 2 or more, TKI therapy should be interrupted, and diuretics can relieve fluid retention if present. TKI should be resumed after effusion resolution with dose reduction in case of further occurrences. If patients with Class 2 or more are symptomatic or with Class 3 or more but asymptomatic, dasatinib therapy should be interrupted and then resumed after the effusion is resolved. However, it should be discontinued if the pleural effusion recurs. In addition, corticosteroids such as 40 mg of prednisone per day, should be given for 4 days, and the pleural fluid should be examined for other potential causes. Another classification approach defines small effusion as a volume of less than 500 ml with blunting costophrenic angle; medium effusion as with opacity above the costophrenic angle; and large effusion when having more than 30–50% of hemithorax. The respective management and monitoring of each severity are presented in Figure 5 (5).

### Pulmonary arterial hypertension

#### Occurrence and consequences

Pulmonary arterial hypertension has been reported with dasatinib therapy, rarely with bosutinib, nilotinib, or ponatinib, whereas no reports with imatinib (3). In PAH, there is usually an increase in pulmonary vascular resistance (PVR) and pulmonary artery pressure (PAP) leading to right ventricular failure (7); mean PAP is above 20 mmHg and PVR is 3 Wood units (WU) or above with normal left cardiac filling pressures (i.e., pulmonary artery wedge pressure (PCWP) ≤15 mmHg) (3, 52). Dasatinib is implicated in increasing the pressure in the pulmonary arterial system, hence causes a pre-capillary PAH (i.e., Clinical Group 1) (7). Earlier, a study using data from the French pulmonary hypertension registry identified nine dasatinib-treated patients with pulmonary hypertension, from dasatinib approval from November 2006 to September 2010. The estimated incidence of dasatinib-related pulmonary hypertension in France was 0.45%. There were not any incidental pulmonary hypertension cases related to other TKIs at the time of diagnosis. Four months after the discontinuation of dasatinib, there were hemodynamic, clinical, and functional improvements. However, there was not a complete recovery in most of the patients after 9 months of follow-up (20). Another study from the same French registry investigated the long-term outcomes of 21 patients with dasatinib-induced PAH which was confirmed by right-heart catheterization. Nineteen patients with CML received dasatinib for a median of 42 months before PAH diagnosis. Dasatinib was discontinued in all patients, and half of them (*n* = 11) received therapy for PAH such as bosentan, sildenafil, and calcium channel blockers. Despite improvement after drug discontinuation, PAH continued in 37% of patients. Concomitant pleural and pericardial effusions were detected in 62 and 29% of patients, respectively (53). A multicenter study from Australia retrospectively studied 212 patients with chronic phase CML. The study estimated an occurrence of 5% for dasatinib-induced PAH which was frequently associated with pleural effusion. PAH was reversible in most of the cases after dasatinib discontinuation. Permanent discontinuation of dasatinib in immunologically competent patients was not necessary (48).

Cases with reversible PAH may have an intense pulmonary arterial vasoconstriction (3), while the frequently reported cases with the persistence of PAH may suggest an irreversible pulmonary arterial remodeling (3, 7). The latter proved valid with histological examination of explanted lungs from a patient who required lung transplantation. The survival of patients with dasatinib-induced PAH (i.e., 90.5 and 85.7% at 1- and 5-year follow-up, respectively) was similar to that reported in dasatinib randomized trials in CML (3). PAH, however, is considered a life-threatening consequence of dasatinib long-term therapy, particularly when accompanied by pleural effusion. If left untreated, it may cause right ventricular failure (5). Finally, although ponatinib was associated with a 6% increase in venous thromboembolic events, there were no cases reported for thromboembolic PAH due to TKIs use (3).

#### Management

The incidence of symptomatic PAH is relatively low and does not warrant PAH screening in asymptomatic patients (3). Dasatinib is the TKI with the potential to cause PAH. Early PAH diagnosis and TKI therapy discontinuation are recommended (5). The symptoms of PAH are usually not specific such as dyspnea, fatigue, atypical chest pain, or unexplained syncope. The presence of non-specific symptoms should raise suspicion about PAH diagnosis and prompt investigation using chest X-ray and echocardiogram with Doppler flow, as well as considering referral to cardiology service if warranted. A baseline echocardiogram before dasatinib initiation may be performed, if feasible, to identify a pre-existing PAH (3). If PAH is suspected during TKI therapy, a chest X-ray is performed to rule out pleural effusion, and an echocardiogram is performed to assess PAP and then identify the cause of the elevated PAP (≥25 mmHg). A right-heart catheterization is indicated to confirm PAH diagnosis (5, 7, 18).

**Figure 6:**
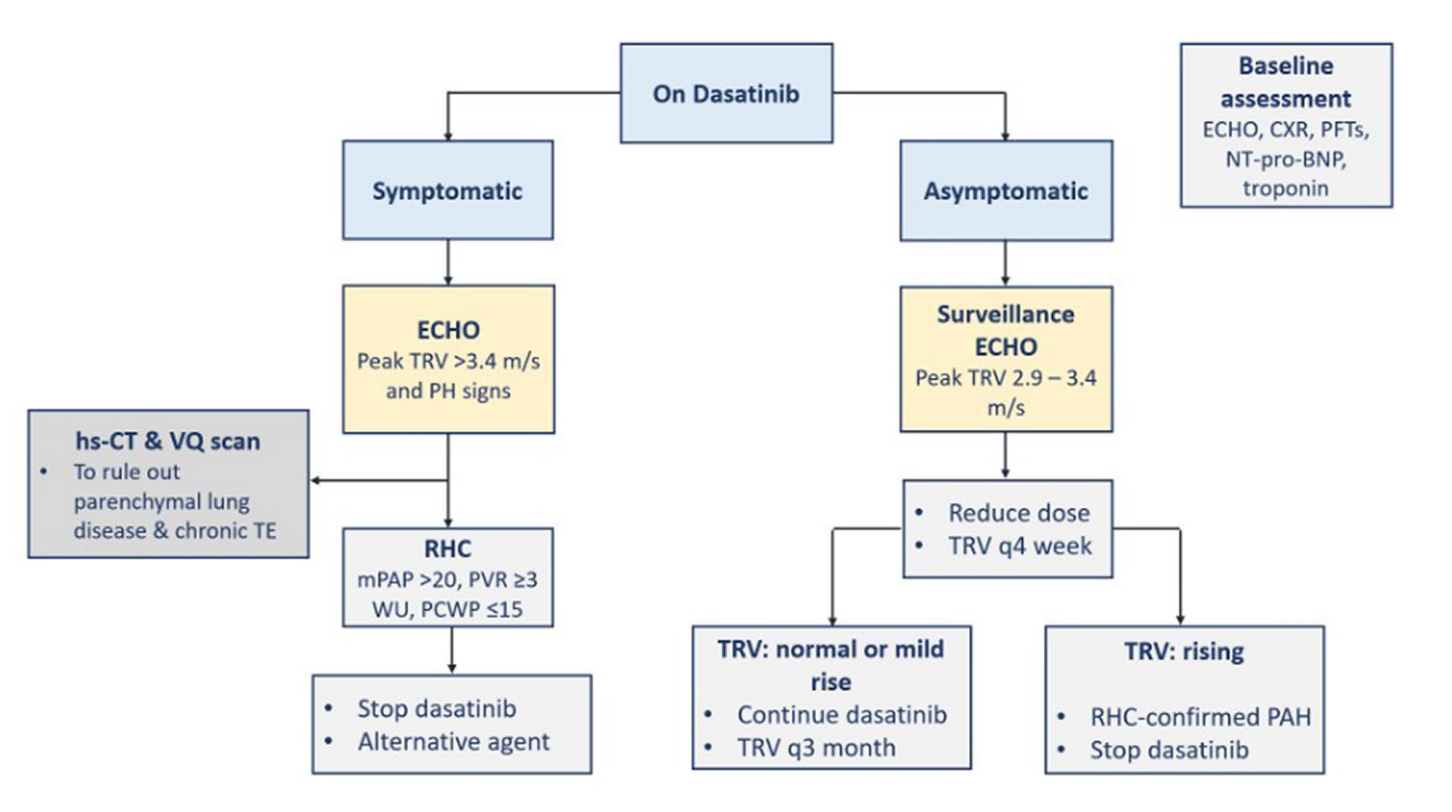
Diagnosis and management of TKI-induced PAH. BNP, B-type natriuretic peptide; CXR, chest x-ray; ECHO, echocardiography; hs-CT, high resolution computed tomography; mPAP, mean pulmonary arterial pressure; PAH, pulmonary arterial hypertension; PCWP, pulmonary artery wedge pressure; PFTs, pulmonary function tests; PH, pulmonary hypertension; PVR, pulmonary vascular resistance; RHC, right heart catheterization; TE, thromboembolism; TKI, tyrosine kinase inhibitor; TRV, tricuspid regurgitation velocity; VQ, ventilation perfusion; WU, Wood units.

When there is a high probability of a new pulmonary hypertension occurrence in a CML patient (i.e., peak tricuspid regurgitation velocity (TRV) is more than 3.4 m/s which is equivalent to an estimated PAP of 50 mmHg or more), TKI therapy should be discontinued until ruled out or confirmed with right-heart catheterization (22). More specifically, if dasatinib-induced PAH is diagnosed, dasatinib therapy should be discontinued (5, 7, 22), and alternative TKI is recommended (22). If dasatinib-treated patients have new asymptomatic peak TRV between 2.9 and 3.4 m/s, the dasatinib dose can be reduced and peak TRV should be monitored every 4 weeks with echocardiography. If peak TRV keeps rising and PAH is confirmed by right-heart catheterization, dasatinib should be stopped (22). Symptoms and PAP usually improve after dasatinib discontinuation (5, 7) but usually without complete recovery of the hemodynamic parameters (5). The overall treatment of pulmonary hypertension should be according to the respective societal guidelines (22). The management of PAH is directed toward the FDA-approved drug classes for PAH treatment and collaboration with the PAH team (5, 7). FDA-approved drug classes include phosphodiesterase type-5 inhibitors (e.g., sildenafil and tadalafil), endothelin receptor-1 antagonists (e.g., bosentan, ambrisentan, and macitentan), prostacyclin derivatives (e.g., systemic epoprostenol, inhaled iloprost, and oral and systemic treprostinil), soluble guanylate cyclase stimulator (e.g., oral riociguat), and prostacyclin receptor agonist (oral selexipag) (5). The steps for the diagnosis and management of TKI-induced PAH are demonstrated in Figure 6 (3, 5, 7, 22). There is not any evidence suggesting the effectiveness of calcium channel blockers in dasatinib-induced PAH (5).

### Drug–drug interactions

Most of the TKIs are metabolized by CYP enzymes and transported by P-glycoprotein (P-gp) (2, 7). Strong CYP enzyme inhibitors can increase TKIs plasma concentrations (1, 16), and vice versa as TKIs can inhibit CYP enzymes to varying degrees (7). Dasatinib can also influence P-gp, the drug transporter (2). Table 6 lists the drug–drug interactions between TKI agents and the most used drugs in cardiopulmonary conditions (54).

## Summary

The introduction of TKIs is considered a milestone in the treatment of CML but their use was associated with a range of serious cardiopulmonary toxicities including vascular adverse events, QT prolongation, heart failure, pleural effusion, and PAH. The current fully approved TKIs (imatinib, bosutinib, dasatinib, nilotinib, and ponatinib) have different efficacy and safety profiles. The exact underlying mechanisms for cardiopulmonary toxicities are not fully known but on- and off-target effects may be partly responsible. Imatinib has the most favorable safety profile among TKIs. Nilotinib and ponatinib were associated with vascular occlusive adverse events, whereas dasatinib was with pleural effusion and PAH. QT prolongation was mostly linked to third-generation agents. TKI-induced adverse events can lead to serious morbidities if left untreated.

The assessment of cardiovascular risk is crucial before starting TKI therapy. The choice of TKI agent should be dictated by its safety profile, disease state, patient comorbidities, and other concurrent therapies considering their interacting tendency. The management of cardiopulmonary adverse events should be integrated with a multidisciplinary collaboration between the respective specialties. Early recognition, frequent monitoring, optimal intervention, and adequate follow-up are essential to managing adverse events while maintaining the long-term TKI therapy benefit. Awareness of the potential TKIs toxicities and balancing the risks and benefits of such therapy should be considered. Future research should fill in the gaps in knowledge about molecular mechanisms, frequencies, definitions, classifications, predictors, and management guidelines of TKIs toxicities.

## Author contributions

All authors listed have made a substantial, direct, and intellectual contribution to the work and approved it for publication. All authors contributed equally to the manuscript.

## Funding

This work was supported by the Academic Health System, Hamad Medical Corporation.

## Conflict of interest

RK, WD, KA, and MY were employed by the Hamad Medical Corporation.

## Publisher’s note

All claims expressed in this article are solely those of the authors and do not necessarily represent those of their affiliated organizations, or those of the publisher, the editors and the reviewers. Any product that may be evaluated in this article, or claim that may be made by its manufacturer, is not guaranteed or endorsed by the publisher.

## Glossary

Acronym: Explanation
ABI: Ankle-brachial index
CML: Chronic myeloid leukemia
CRCD: Chemotherapy-related cardiac dysfunction
CTCAE: Common Terminology Criteria for Adverse Events
CTRCD: Cancer therapy-related cardiac dysfunction
CYP: Cytochrome P450
DAVLEC: DAsatinib, very low-dose, for elderly CML-CP patients
ECG: Electrocardiogram
EGFR: Epidermal growth factor receptor
ENESTnd: Evaluating Nilotinib Efficacy and Safety in Clinical Trials–Newly Diagnosed Patients
FDA: Food and Drug Administration
FEARS: Food and Drug Administration adverse event reporting system
GLS: Global longitudinal strain
hERG: Human Ether-a-go-go
LVEF: Left ventricular ejection fraction
NCI: National Cancer Institute
OPTIC: Optimizing Ponatinib Treatment in CP-CML
OR: Odds ratio
PAH: Pulmonary arterial hypertension
PAOD: Peripheral arterial occlusive disease
PAP: Pulmonary artery pressure
PDGFR: Platelet-derived growth factor receptor
QTc: Corrected QT interval
ROCK: Rho-associated coiled-coil containing kinase
TKI(s): Tyrosine kinase inhibitor(s)
VEGFR: Vascular endothelial growth factor receptor

## Abbreviations

BNP: B-type natriuretic peptide
CXR: chest x-ray
ECHO: echocardiography
hs-CT: high resolution computed tomography
mPAP: mean pulmonary arterial pressure
PAH: pulmonary arterial hypertension
PCWP: pulmonary artery wedge pressure
PFTs: pulmonary function tests
PH: pulmonary hypertension
PVR: pulmonary vascular resistance
RHC: right heart catheterization
TE: thromboembolism
TKI: tyrosine kinase inhibitor
TRV: tricuspid regurgitation velocity
VQ: ventilation perfusion
WU: wood units
